# Knowledge, Attitude and Practice About Artificial Intelligence Among Medical Undergraduate and Postgraduate Students in a Tertiary Care Centre: A Cross-Sectional Survey

**DOI:** 10.7759/cureus.100576

**Published:** 2026-01-01

**Authors:** Sumit Prasad, Anant Khot, Ganesh Dakhale

**Affiliations:** 1 Pharmacology, All India Institute of Medical Sciences, Nagpur, IND

**Keywords:** artificial intelligence (ai) in medicine, crosssectional study, kap study, medical education, medical students

## Abstract

Background

Artificial intelligence (AI) in medicine is already used in many areas such as medical imaging, diagnosis, treatment planning, and drug development. Teaching and formal training about AI in medical schools is limited, especially in low- and middle-income countries. There is little evidence on what medical students know about AI, how they feel about it, and how they actually use it in study and research. Hence, this study assessed knowledge, attitudes, and practices related to AI among undergraduate and postgraduate students at a tertiary care teaching hospital.

Methods

A cross-sectional study was conducted at All India Institute of Medical Sciences (AIIMS), Nagpur, among undergraduate (MBBS) and postgraduate (MD/MS) students, with a total of 317 students. A structured, pre-validated questionnaire was administered using a hard copy. The survey comprised four sections: demographic details, knowledge, attitude, and practice.

Results

A total of 317 medical students participated, with 188 (59.3%) undergraduates and 129 (40.7%) postgraduates. Most had basic knowledge of AI, though few had formal training. Attitudes were largely positive, with strong support for including AI in medical education and awareness of ethical issues. Students reported frequent use of AI in study, research, and brainstorming, but less for career guidance.

Conclusion

Medical students have moderate knowledge of AI and positive attitudes toward its use in healthcare, but actual use is low due to limited training, concerns about accuracy, and lack of integration, highlighting the need for focused education and practical exposure to AI tools in medical training.

## Introduction

Artificial intelligence (AI) is broadly defined as "a system's ability to correctly interpret external data, to learn from such data, and to use those learnings to achieve specific goals and tasks through flexible adaptation" [1,2]. Over the past decade, AI has emerged as a transformative force across diverse domains, including computer science, industrial automation, finance, law, and scientific research [3,4]. In the field of healthcare, AI technologies are increasingly being integrated into clinical workflows and decision-making processes. Applications range from medical imaging and diagnostic support to drug discovery and personalized treatment planning. Notably, AI has demonstrated utility in radiology, neurosurgical imaging, dermatological assessments, tumor classification, management of chest pain, and early diagnosis of neurological disorders such as Alzheimer's disease [5-7]. AI is also being used to guide therapy selection, particularly in patients with multiple comorbidities and complex treatment regimens, as well as in stratified care delivery, and it can also offer cost-effective solutions to a variety of health challenges, thereby improving accessibility and efficiency in healthcare systems [5,8].

Despite this growing integration of AI in clinical practice, its incorporation into medical education remains inconsistent. Medical students and trainees, who represent the future healthcare workforce, must be adequately prepared to engage with AI-driven tools. However, there is limited empirical evidence on their knowledge, attitudes, and practices (KAP) related to AI, particularly in low- and middle-income countries (LMICs). Hence, this study is planned to address this gap by assessing the KAP regarding AI among undergraduate and postgraduate medical students in a tertiary care teaching hospital.

## Materials and methods

Study design and setting

This was a cross-sectional, questionnaire-based study conducted by the Department of Pharmacology at the All India Institute of Medical Sciences (AIIMS), Nagpur. The study targeted undergraduate (MBBS) students from the second phase onwards and postgraduate (MD/MS) students from all professional years, conducted over a four-month period, from December 2024 to April 2025. Convenience sampling was employed, where all eligible participants present during the data collection period were invited to participate. Participation was voluntary. Students who provided informed consent and submitted complete responses were included in the study.

Ethical considerations

The study was initiated after receiving clearance from the Institutional Ethics Committee of AIIMS, Nagpur (IEC/Pharmac/2024/1030). Administrative clearance was also secured from the institutional leadership. The protocol adhered to the ethical principles outlined in the Declaration of Helsinki. Confidentiality was ensured throughout the data collection and analysis process.

Data collection processes

Participants were briefed about the purpose and scope of the study and administered a structured, pre-validated questionnaire [3] in hard-copy format. The questionnaire comprised four sections: demographic details, knowledge related to AI, attitudes towards AI in medicine, and self-reported practices regarding AI use. A time frame of 15 minutes was allotted for the questionnaire, completion under supervision by the investigator to ensure independent responses.

Structure of the questionnaire

The question included the following sections.

Demographic Details

This section comprises participants' initial, age, sex (male/female), educational level (UG/PG), graduation year (1st/2nd/3rd), and specialty.

Knowledge

Seven items assessed knowledge about AI as dichotomous (yes/no) responses, and two open-ended questions assessed the applications and barriers.

Attitude

Attitudes were measured by 10 items on a five-point Likert scale (from strongly disagree to strongly agree), assessing beliefs regarding AI implementation in education, research, and clinical practice.

Practice

Six items measured practices on a five-point Likert scale (from never to all the time), assessing the uses of AI in different activities such as personal development, research, grammar check, exam preparations, etc.

Sample size calculation

Convenience sampling was done in the present study [9]. Assuming that 71% [3] of the subjects in the population have the factor of interest, the study would require a minimum sample size of 317 for estimating the expected proportion with 5% absolute precision and 95% confidence.

Data analysis

Quantitative variables were summarized using means and standard deviations, while categorical variables were presented as frequencies and percentages. Associations between selected categorical variables were assessed using the Chi-square test. Data entry and analysis were performed using Microsoft Excel (Microsoft Corp., Redmond, US).

## Results

A total of 450 hard-copy questionnaires were distributed to students, of which 317 (70.4%) completed questionnaires were returned and included in the analysis. Among the respondents, 188 (59.3%) were undergraduates and 129 (40.7%) were postgraduates, representing 27 medical specialties. The highest proportion of postgraduate participants was from general medicine and obstetrics and gynecology. The mean age of the participants was 22.7±3.7 years. Gender information was provided by 315 respondents, of whom 186 (58.7%) were male, and 131 (41.3%) were female.

Knowledge

Most participants, 239 of 317 (75.4%), reported having basic knowledge of artificial intelligence. Only 83 of 316 (26.3%) indicated they knew what deep learning or machine learning is. A little over half, 172 of 310 (55.5%), said they knew at least one application of AI in their field. Formal instruction and training were uncommon: 31 of 317 (9.8%) had attended any AI course, and 38 of 317 (12%) had been taught about AI during undergraduate studies. Two-thirds, 202 of 306 (66%), agreed that AI requires a lot of labelled data to learn. Less than half, 139 of 307 (45.3%), reported that they understood the barriers to applying AI in medicine.

**Table 1:** Knowledge of artificial intelligence among healthcare students

Item (n), response		n	%
Do you have knowledge of the basics of AI? (317)	Yes	239	75.4%
	No	78	24.6%
Do you know what deep learning/machine learning is? (316)	Yes	83	26.3%
	No	233	73.7%
Do you know any application of AI in your field of interest? (310)	Yes	172	55.5%
	No	138	44.5%
Have you attended any previous online/offline courses regarding AI? (317)	Yes	31	9.8%
	No	286	90.2%
Have you ever been taught about AI in your undergraduate studies? (317)	Yes	38	12%
	No	279	88%
AI requires a lot of labelled data to learn (data already processed by a human) (306)	Yes	202	66%
	No	104	34%
I understand the barriers of applying AI in medicine (307)	Yes	139	45.3%
	No	168	54.7%

Only one item showed a statistically significant difference between males and females. Knowledge of machine learning/deep learning was higher among males (31.15%) compared with females (19.85%), with a statistically significant difference (p=0.025). To explore differences in AI-related awareness and exposure across educational levels, responses were compared between undergraduate (UG) and postgraduate (PG) students (see Table 2). 

**Table 2:** Comparison of AI knowledge between undergraduate and postgraduate students

Item, response n (%)	Undergraduates (UG)	Postgraduates (PG)	p-value
Do you have knowledge of the basics of AI? (317)			0.84
Yes	141 (75%)	98 (75.97%)	
No	47 (25%)	31 (24.03%)	
Do you know what deep learning/machine learning is? (316)			0.35
Yes	53 (28.19%)	30 (23.44%)	
No	135 (71.81%)	98 (76.56%)	
Do you know any application of AI in your field of interest? (310)			0.001
Yes	87 (47.8%)	85 (66.41%)	
No	95 (52.2%)	43 (33.59%)	
Have you attended any previous online/offline courses regarding AI? (317)			0.004
Yes	11 (5.85%)	20 (15.5%)	
No	177 (94.15%)	109 (84.5%)	
Have you ever been taught about AI in your undergraduate studies? (317)			0.054
Yes	28 (14.89%)	10 (7.75%)	
No	160 (85.11%)	119 (92.25%)	
AI requires a lot of labelled data to learn (data already processed by a human) (306)			0.84
Yes	120 (65.57%)	82 (66.67%)	
No	63 (34.43%)	41 (33.33%)	
I understand the barriers of applying AI in medicine (307)			0.13
Yes	75 (41.67%)	64 (50.39%)	
No	105 (58.33%)	63 (49.61%)	

Attitude

Students were broadly positive about learning AI and about its ethical implications. For the statement that healthcare students should learn the basics of AI, 281 of 316 respondents (88.9%) either agreed or strongly agreed, 25 (7.9%) were neutral, and 10 (3.2%) disagreed or strongly disagreed. For the view that AI will be a highly required tool in their field, 244 of 315 (77.5%) agreed or strongly agreed, 51 (16.2%) were neutral, and 20 (6.3%) disagreed or strongly disagreed. For the need to understand ethical implications, 291 of 316 (92.1%) agreed or strongly agreed, 18 (5.7%) were neutral, and 7 (2.2%) disagreed or strongly disagreed.

A majority also agreed that AI will change the educational system: 249 of 317 (78.5%) agreed or strongly agreed, 58 (18.3%) were neutral, and 10 (3.2%) disagreed or strongly disagreed. However, only 52 of 316 (16.5%) agreed or strongly agreed that human teachers will be replaced in the foreseeable future; 76 (24.1%) were neutral, and 188 (59.5%) disagreed or strongly disagreed. Regarding excitement about upcoming educational developments, 223 of 317 (70.3%) agreed or strongly agreed, 79 (24.9%) were neutral, and 15 (4.7%) disagreed or strongly disagreed.

Most participants supported including AI in medical training: 256 of 316 (81.0%) agreed or strongly agreed, 46 (14.6%) were neutral, and 14 (4.4%) disagreed or strongly disagreed. Only 33 of 317 (10.4%) agreed or strongly agreed that clinical AI will be more accurate than physicians; 85 (26.8%) were neutral, and 199 (62.8%) disagreed or strongly disagreed. On whether some specialties are more prone to being replaced by AI than others, 180 of 315 (57.0%) agreed or strongly agreed, 79 (25.0%) were neutral, and 57 (18.0%) disagreed or strongly disagreed. Finally, 89 of 316 (28.2%) agreed or strongly agreed that AI would increase diagnostic errors, 122 (38.6%) were neutral, and 105 (33.2%) disagreed or strongly disagreed.

**Table 3:** Attitudes of healthcare students towards artificial intelligence

Item (n), response		n	%
I believe healthcare students should learn the basics of AI (316)	Strongly agree	139	44.0%
	Agree	142	44.9%
	Neutral	25	7.9%
	Disagree	3	0.9%
	Strongly disagree	7	2.2%
I believe AI will be a highly required tool in my field (315)	Strongly agree	103	32.7%
	Agree	141	44.8%
	Neutral	51	16.2%
	Disagree	16	5.1%
	Strongly disagree	4	1.3%
I believe ethical implications of AI must be understood by different students (316)	Strongly agree	146	46.2%
	Agree	145	45.9%
	Neutral	18	5.7%
	Disagree	3	0.9%
	Strongly disagree	4	1.3%
I believe AI will revolutionize the educational system (317)	Strongly agree	106	33.4%
	Agree	143	45.1%
	Neutral	58	18.3%
	Disagree	5	1.6%
	Strongly disagree	5	1.6%
I believe human teachers will be replaced in the foreseeable future (316)	Strongly agree	23	7.3%
	Agree	29	9.2%
	Neutral	76	24.1%
	Disagree	124	39.2%
	Strongly disagree	64	20.3%
I believe the upcoming developments in the educational system will excite me (317)	Strongly agree	69	21.8%
	Agree	154	48.6%
	Neutral	79	24.9%
	Disagree	7	2.2%
	Strongly disagree	8	2.5%
I believe AI should be a part of the training system among students of medical fields (316)	Strongly agree	89	28.2%
	Agree	167	52.8%
	Neutral	46	14.6%
	Disagree	11	3.5%
	Strongly disagree	3	0.9%
Clinical AI will be more accurate than physicians (317)	Strongly agree	15	4.7%
	Agree	18	5.7%
	Neutral	85	26.8%
	Disagree	127	40.1%
	Strongly disagree	72	22.7%
I believe some specialties are more prone to be replaced by AI than others (316)	Strongly Agree	61	19.3%
	Agree	119	37.7%
	Neutral	79	25.0%
	Disagree	41	13.0%
	Strongly disagree	16	5.1%
I believe AI would increase the percentage of errors in diagnosis (316)	Strongly agree	31	9.8%
	Agree	58	18.4%
	Neutral	122	38.6%
	Disagree	87	27.5%
	Strongly disagree	18	5.7%

Two items showed statistically significant gender differences. A higher proportion of females agreed that healthcare students should learn the basics of AI (p=0.044). Females also expressed stronger agreement that the ethical implications of AI must be understood by students (p=0.017). The differences in AI-related attitude across educational levels, responses were compared between undergraduate and postgraduate students (see Table 4).

**Table 4:** Comparison of attitudes between undergraduate and postgraduate students

Item, response n (%)	Undergraduates (UG)	Postgraduates (PG)	p-value
I believe healthcare students should learn the basics of AI (316)			0.51
Strongly agree	84 (44.92%)	55 (42.64%)	
Agree	79 (42.25%)	63 (48.84%)	
Neutral	17 (9.09%)	8 (6.2%)	
Disagree	3 (1.60%)	0 (0%)	
Strongly disagree	4 (2.14%)	3 (2.33%)	
I believe AI will be a highly required tool in my field (315)			0.002
Strongly agree	61 (32.62%)	42 (32.81%)	
Agree	73 (39.04%)	68 (53.13%)	
Neutral	38 (20.32%)	13 (10.16%)	
Disagree	14 (7.49%)	2 (1.56%)	
Strongly disagree	1 (0.53%)	3 (2.34%)	
I believe ethical implications of AI must be understood by different students (316)			<0.001
Strongly agree	85 (45.45%)	61 (47.29%)	
Agree	78 (41.71%)	67 (51.94%)	
Neutal	17 (9.09%)	1 (0.78%)	
Disagree	3 (1.60%)	0 (0%)	
Strongly disagree	4 (2.14%)	0 (0%)	
I believe AI will revolutionize the educational system (317)			0.42
Strongly agree	58 (30.85%)	48 (37.21%)	
Agree	83 (44.15%)	60 (46.51%)	
Neutral	39 (20.74%)	19 (14.73%)	
Disagree	4 (2.13%)	1 (0.78%)	
Strongly disagree	4 (2.13%)	1 (0.78%)	
I believe human teachers will be replaced in the foreseeable future (316)			0.14
Strongly agree	17 (9.04%)	6 (4.69%)	
Agree	17 (9.04%)	12 (9.38%)	
Neutral	47 (25.00%)	29 (22.66%)	
Disagree	64 (34.04%)	60 (46.88%)	
Strongly disagree	43 (22.87%)	21 (16.41%)	
I believe the upcoming developments in the educational system will excite me (317)			0.063
Strongly agree	34 (18.09%)	35 (27.13%)	
Agree	89 (47.34%)	65 (50.39%)	
Neutral	55 (29.26%)	24 (18.60%)	
Disagree	6 (3.19%)	1 (0.78%)	
Strongly disagree	4 (2.13%)	4 (3.10%)	
I believe AI should be a part of the training system among students of medical fields (316)			0.45
Strongly agree	49 (26.06%)	40 (31.25%)	
Agree	97 (51.6%)	70 (54.69%)	
Neutral	32 (17.02%)	14 (10.94%)	
Disagree	8 (4.26%)	3 (2.34%)	
Strongly disagree	2 (1.06%)	1 (0.78%)	
Clinical AI will be more accurate than physicians (317)			0.15
Strongly agree	9 (4.79%)	6 (4.65%)	
Agree	14 (7.45%)	4 (3.1%)	
Neutral	56 (29.79%)	29 (22.48%)	
Disagree	73 (38.83%)	54 (41.86%)	
Strongly disagree	36 (19.15%)	36 (27.91%)	
I believe some specialties are more prone to be replaced by AI than others (316)			0.45
Strongly agree	30 (16.04%)	31 (24.03%)	
Agree	73 (39.04%)	46 (35.66%)	
Neutral	47 (25.13%)	32 (24.81%)	
Disagree	26 (13.9%)	15 (11.63%)	
Strongly disagree	11 (5.88%)	5 (3.88%)	
I believe AI would increase the percentage of errors in diagnosis (316)			0.016
Strongly agree	20 (10.7%)	11 (8.53%)	
Agree	24 (12.83%)	34 (26.36%)	
Neutral	83 (44.39%)	39 (30.23%)	
Disagree	50 (26.74%)	37 (28.68%)	
Strongly disagree	10 (5.35%)	8 (6.20%)	

Practice

The use of AI in study and research was common. For preparing exams and assignments, 195 of 317 respondents (61.5%) reported using AI often, most of the time, or all the time; 96 (30.3%) used it rarely, and 26 (8.2%) never. For research, 201 of 317 (63.4%) used AI often, most of the time, or all the time; 80 (25.2%) rarely, and 36 (11.4%) never. For idea generation and brainstorming, 215 of 317 (67.8%) used AI often or more, 79 (24.9%) rarely, and 23 (7.3%) never.

Use of AI for personal choices or career guidance was less frequent: 119 of 316 (37.7%) used it often, most of the time, or all the time; 130 (41.1%) rarely, and 67 (21.2%) never. For spelling and grammar checking, 171 of 317 (53.9%) used AI often or more, 87 (27.4%) rarely, and 59 (18.6%) never. For personality development and other skills, 130 of 317 (41.0%) used AI often or more, 119 (37.5%) rarely, and 68 (21.5%) never.

**Table 5:** Practice and usage of artificial intelligence by healthcare students

Item (n), response		n	%
How frequently do you use AI to prepare for your exams? How frequently do you use AI to prepare for your homework/assignments (317)	Never	26	8.2%
	Rarely	96	30.3%
	Often	118	37.2%
	Most of the time	63	19.9%
	All the time	14	4.4%
How frequently do you use AI to conduct your research (317)	Never	36	11.4%
	Rarely	80	25.2%
	Often	135	42.6%
	Most of the time	54	17.0%
	All the time	12	3.8%
How frequently do you use AI for idea generation and brainstorming (317)	Never	23	7.3%
	Rarely	79	24.9%
	Often	137	43.2%
	Most of the time	67	21.1%
	All the time	11	3.5%
How frequently do you use AI for personal choices/career guidance (316)	Never	67	21.2%
	Rarely	130	41.1%
	Often	80	25.3%
	Most of the time	33	10.4%
	All the time	6	1.9%
How frequently do you use AI for spelling and grammar checking (317)	Never	59	18.6%
	Rarely	87	27.4%
	Often	96	30.3%
	Most of the time	53	16.7%
	All the time	22	6.9%
How frequently do you use AI for personality development and other skills (317)	Never	68	21.5%
	Rarely	119	37.5%
	Often	100	31.5%
	Most of the time	19	6.0%
	All the time	11	3.5%

Only two items showed significant gender differences. Males reported using AI for research more frequently than females (p=0.036). Females reported using AI for spelling and grammar checking more often than males (p=0.006). The differences in AI-related practice across educational levels were compared between undergraduate and postgraduate students (see Table 6).

**Table 6:** Comparison of practices between undergraduate and postgraduate students

Item, response n (%)	Undergraduates (UG)	Postgraduate (PG)	p-value
How frequently do you use AI to prepare for your exams? How frequently do you use AI to prepare for your homework/assignments (317)			0.005
Never	14 (7.45%)	12 (9.3%)	
Rarely	44 (23.40%)	52 (40.31%)	
Often	82 (43.62%)	36 (27.91%)	
Most of the time	37 (19.68%)	26 (20.16%)	
All the time	11 (5.85%)	3 (2.33%)	
How frequently do you use AI to conduct your research (317)			0.013
Never	26 (13.83%)	10 (7.75%)	
Rarely	42 (22.34%)	38 (29.46%)	
Often	78 (41.49%)	57 (44.19%)	
Most of the time	30 (15.96%)	24 (18.6%)	
All the time	12 (6.38%)	0 (0%)	
How frequently do you use AI for idea generation and brainstorming (317)			0.65
Never	16 (8.51%)	7 (5.43%)	
Rarely	44 (23.4%)	35 (27.13%)	
Often	82 (43.62%)	55 (42.64%)	
Most of the time	38 (20.21%)	29 (22.48%)	
All the time	8 (4.26%)	3 (2.33%)	
How frequently do you use AI for personal choices/career guidance (316)			<0.001
Never	55 (29.26%)	12 (9.38%)	
Rarely	65 (34.57%)	65 (50.78%)	
Often	42 (22.34%)	38 (29.69%)	
Most of the time	24 (12.77%)	9 (7.03%)	
All the time	2 (1.06%)	4 (3.13%)	
How frequently do you use AI for spelling and grammar checking (317)			<0.001
Never	48 (25.53%)	11 (8.53%)	
Rarely	40 (21.28%)	47 (36.43%)	
Often	56 (29.79%)	40 (31.01%)	
Most of the time	34 (18.09%)	19 (14.73%)	
All the time	10 (5.32%)	12 (9.3%)	
How frequently do you use AI for personality development and other skills (317)			0.013
Never	50 (26.6%)	18 (13.95%)	
Rarely	62 (32.98%)	57 (44.19%)	
Often	56 (29.79%)	44 (34.11%)	
Most of the time	15 (7.98%)	4 (3.1%)	
All the time	5 (2.66%)	6 (4.65%)	

## Discussion

This study aimed to evaluate the KAP related to AI in medicine among undergraduate and postgraduate medical students in a tertiary care teaching hospital. Our findings are aligned with international research showing that medical trainees have a generally positive view of AI but lack formal education on the subject. For example, Al Hadithy et al. reported that most clinical-year medical students in Oman viewed AI favorably, even though 75% had never directly encountered AI in healthcare. These students strongly supported the inclusion of AI training in the medical curriculum [10]. Similarly, a recent study in China found that over 60% of medical students had used ChatGPT for academic purposes, such as gathering information and completing assignments, and about the same proportion expressed optimism about the role of AI in medicine [11].

In our study, 59.9% of participants agreed that AI will play a vital role in future healthcare, and a substantial proportion reported using AI tools such as ChatGPT and Gemini Grok, with 35.1% using them frequently. This mirrors global trends where students are increasingly engaging with AI technologies for learning and research. However, concerns about misinformation and plagiarism remain widespread, highlighting the need for clear guidelines on the responsible use of these tools [11]. Interestingly, only a small number of respondents in our study felt that AI could fully replace physicians, indicating that most students see AI as an aid to, rather than a replacement for, human decision-making [12].

Despite high interest and positive attitudes, our findings revealed significant gaps in knowledge. Only 41.5% of participants answered core AI-related questions correctly. This is consistent with other studies that have reported similar trends. For instance, a large survey in Syria found that only 12% of respondents demonstrated strong knowledge of AI concepts [12]. Likewise, the Omani study showed a median knowledge score of just 3.25 out of 5, with little variation across academic years [10]. These results suggest that while students recognize the importance of AI, their understanding of its principles remains limited without structured teaching. Several authors have emphasized the importance of integrating AI into medical curricula so that future clinicians are equipped to understand its applications and limitations [10,11]. 

One strength of our study is that it included both undergraduate and postgraduate students, whereas many previous studies focused on a single group. This broader approach allowed us to compare perceptions across different levels of training and explore whether seniority or clinical experience influenced familiarity with AI. Our sample size of 317 provided sufficient power to identify subgroup differences, such as those between years of study or specialties. Prior research suggests that more advanced students may report slightly greater confidence with AI, possibly due to increased exposure during clinical training [13].

In addition, our survey included questions about barriers to AI adoption. Many participants expressed concerns about data privacy, lack of technical support, and unclear policies, similar to barriers reported in other settings [13]. A study from Pakistan found that these challenges were common and often stemmed from limited access to technology, insufficient institutional support, and a lack of trained faculty to teach AI [13]. Our results indicate that these systemic barriers could hinder the effective integration of AI into medical education and practice.

This study has some limitations. The use of convenience sampling may have led to the over-representation of students who are more interested in technology, potentially inflating overall KAP levels. Additionally, structured questionnaires, while useful for collecting quantitative data, cannot fully capture the complexity of students' attitudes, particularly regarding ethical concerns and practical applications of AI. The absence of a qualitative component, such as focus groups or interviews, limited our ability to explore the underlying reasons for students' views. Future research could benefit from mixed methods designs to provide deeper insights into these perspectives.

Our results and those from other studies indicate that medical students are eager to engage with AI but lack the necessary knowledge and training to use it effectively. Educational institutions should consider formally incorporating AI concepts and ethical considerations into medical curricula to prepare future healthcare providers for its growing role [10,11]. Institutions should also invest in infrastructure, faculty training, and clear policies to guide AI use [13].

The results of the present study suggest that medical students from a wide range of disciplines in India are progressively incorporating AI into their everyday academic work, although the extent and objectives of its use differ markedly. AI-based tools are used predominantly for basic academic functions, including language correction, academic writing, and research-related assistance, while their application in clinical contexts or career-oriented activities remains relatively limited. In the context of the expanding adoption of AI across areas such as diagnostics, biomedical research, pharmacovigilance, and clinical decision support, these findings highlight the importance of systematic integration of AI education into the Indian medical curriculum. Undergraduate and postgraduate training programs should include structured instruction on fundamental AI principles, core data literacy skills, and the ethical and responsible use of digital health technologies. In this regard, regulatory authorities, including the National Medical Commission, may consider articulating competency-based educational objectives that promote critical evaluation of AI-generated information, awareness of algorithmic biases and limitations, and appropriate application of AI-assisted clinical reasoning, consistent with patient safety and evidence-based medical practice.

Future investigations should prioritize probability-based sampling approaches to enhance the representativeness and generalizability of findings across diverse educational and healthcare settings. The incorporation of qualitative methodologies, such as open-ended questionnaires or focus-group discussions, would facilitate a more nuanced understanding of students' attitudes, concerns, and contextual factors influencing AI adoption. Additionally, longitudinal study designs are recommended to assess changes in knowledge, attitudes, and practices as students advance through medical training and encounter real-world AI applications, thereby generating evidence to guide curriculum development and faculty training strategies.

## Conclusions

The present study highlights that medical students, both at undergraduate and postgraduate levels, possess a moderate understanding of AI in the context of healthcare. While their attitudes toward AI were largely positive, particularly regarding its potential to enhance clinical decision-making and efficiency, actual utilization of AI tools remains suboptimal. Key barriers identified include limited integration of AI into existing clinical workflows, concerns about the accuracy and trustworthiness of AI systems, and insufficient real-world validation. These insights suggest that while there is foundational interest and openness toward AI in medicine, significant gaps remain in knowledge, exposure, and practical implementation. To bridge these gaps, targeted educational initiatives and the incorporation of validated, domain-specific AI tools into medical training are essential. Future research should focus on evaluating the impact of such interventions and exploring the role of AI across specific clinical specialties to facilitate meaningful adoption in routine practice.
